# Genome-wide differential expression profiling of lncRNAs and mRNAs associated with early diabetic cardiomyopathy

**DOI:** 10.1038/s41598-019-51872-9

**Published:** 2019-10-25

**Authors:** Tarun Pant, Anuradha Dhanasekaran, Xiaowen Bai, Ming Zhao, Edward B. Thorp, Joseph M. Forbess, Zeljko J. Bosnjak, Zhi-Dong Ge

**Affiliations:** 1 Departments of Medicine, Medical College of Wisconsin, 8701 Watertown Plank Road, Milwaukee, Wisconsin 53226 USA; 2 Departments of Surgery and Pediatrics, Ann & Robert H. Lurie Children’s Hospital of Chicago, Feinberg School of Medicine, Northwestern University, 225 E. Chicago Avenue, Chicago, Illinois 60611 USA; 3 Centre for Biotechnology, Anna University, Chennai, Tamil Nadu 600025 India; 4 Physiology, Medical College of Wisconsin, 8701 Watertown Plank Road, Milwaukee, Wisconsin 53226 USA; 5 Departments of Cell Biology, Neurobiology & Anatomy, Medical College of Wisconsin, 8701 Watertown Plank Road, Milwaukee, Wisconsin 53226 USA; 6 Division of Cardiology, Department of Medicine, Feinberg School of Medicine, Northwestern University, 300 E. Superior Avenue, Chicago, Illinois 60611 USA; 7 Department of Pathology, Feinberg School of Medicine, Northwestern University, 300 E. Superior Avenue, Chicago, Illinois 60611 USA

**Keywords:** Gene regulation, Genetics research

## Abstract

Diabetic cardiomyopathy is one of the main causes of heart failure and death in patients with diabetes. There are no effective approaches to preventing its development in the clinic. Long noncoding RNAs (lncRNA) are increasingly recognized as important molecular players in cardiovascular disease. Herein we investigated the profiling of cardiac lncRNA and mRNA expression in type 2 diabetic db/db mice with and without early diabetic cardiomyopathy. We found that db/db mice developed cardiac hypertrophy with normal cardiac function at 6 weeks of age but with a decreased diastolic function at 20 weeks of age. LncRNA and mRNA transcripts were remarkably different in 20-week-old db/db mouse hearts compared with both nondiabetic and diabetic controls. Overall 1479 lncRNA transcripts and 1109 mRNA transcripts were aberrantly expressed in 6- and 20-week-old db/db hearts compared with nondiabetic controls. The lncRNA-mRNA co-expression network analysis revealed that 5 deregulated lncRNAs having maximum connections with differentially expressed mRNAs were BC038927, G730013B05Rik, 2700054A10Rik, AK089884, and Daw1. Bioinformatics analysis revealed that these 5 lncRNAs are closely associated with membrane depolarization, action potential conduction, contraction of cardiac myocytes, and actin filament-based movement of cardiac cells. This study profiles differently expressed lncRNAs in type 2 mice with and without early diabetic cardiomyopathy and identifies BC038927, G730013B05Rik, 2700054A10Rik, AK089884, and Daw1 as the core lncRNA with high significance in diabetic cardiomyopathy.

## Introduction

Type 2 diabetes mellitus (T2DM) is a global public health problems with rising number of patients^1^. It is one of the principal causes of heart failure, blindness, renal failure, and stroke^2,3^. Mainly due to metabolic disturbance, diabetic myocardium develops local inflammation, necrosis, apoptosis, fibrosis, atherosclerosis, and ventricular hypertrophy^4,5^. These pathological changes developed in diabetic hearts can lead to cardiac dysfunction in the absence of ischemic heart disease and hypertension, termed diabetic cardiomyopathy (DCM)^5,6^. It is evident that DCM is one of the leading causes of heart failure and death in patients with diabetes mellitus^5,7^. Despite extensive research, no approaches can efficiently prevent the development of DCM in the patients^5,8,9^.

Long noncoding RNAs (lncRNAs) are a diverse type of RNA transcripts exceeding 200 nucleotides in length^10–12^. They do not directly encode proteins but can epigenetically regulate the expression of multiple genes^10,13^. It is clear that lncRNAs are expressed in a cell-type and tissue-specific manner in cardiovascular disease^14,15^. This unique expression of lncRNAs provides the avenue for the diagnosis and treatment of cardiovascular disease. Recent studies indicate that lncRNAs play a crucial role in the development of DCM in type 1 diabetes mellitus^4,9,16^. However, the overall profiling of lncRNA expression in the DCM of T2DM has not been reported.

Accordingly we characterized the cardiovascular phenotypes of the T2DM B6.BKS(D)-Lepr^*db*^/J (db/db) mouse, a widely used preclinical model of T2DM with obesity, to identify the db/db mice with early DCM^17,18^. Using the high-throughput microarray, we investigated the genome-wide expression profiling of deregulated lncRNAs and mRNAs in freshly isolated myocardium from db/db mice with and without DCM. The deregulated genes were further analyzed using the Gene Ontology (GO) and the Kyoto Encyclopedia of Genes and Genomes (KEGG) databases to annotate the potential functions of differentially expressed lncRNAs. The lncRNAs-mRNAs co-expression networks were built to explore the relationship between lncRNAs and mRNAs.

## Results

### General characteristics of control and db/db mice

**Figure 1:**
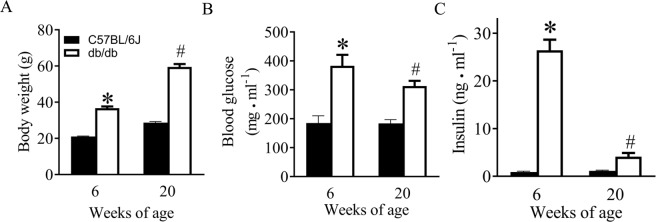
Body weight, blood glucose, and insulin levels of C57BL/6J and db/db mice at 6 and 20 weeks of age. (**A**) body weight; (**B**) blood glucose; and (**C**) plasma insulin levels. Data are presented as means ± SEM. Kruskal-Wallis test followed by Dunn’s test was used to analyze multiple group comparisons. *P < 0.05 versus C57BL/6J group at 6 weeks old; ^#^P < 0.05 versus C57BL/6J group at 20 weeks old (n = 12–13 mice/group).

General characteristics of the db/db and C57BL/6J mice are showed in Fig. 1. Db/db mice at 6 weeks of age were significantly heavier than age-matched C57BL/6J mice (P < 0.05, n = 10 mice/group). Fasting blood glucose levels were significantly higher in db/db than C57BL/6J mice at 6 weeks of age. Body weight, but not blood glucose levels, was further elevated in 20-week-old db/db mice compared with 6-week-old db/db mice. Insulin levels in plasma were significantly higher in db/db mice at 6 weeks of age than age-matched controls. Although insulin levels were significantly lower in 20-week-old db/db mice than 6-week-old db/db ones, they were higher in db/db than C57BL/6J mice at 20 weeks of age (P < 0.05, n = 10 mice/group).

### Left ventricular hemodynamics of control and db/db mice

Table 1 lists left ventricular (LV) hemodynamic parameters of C57BL/6J and db/db mice at 6 and 20 weeks of age. There were no significant differences in heart rate, LV end-systolic pressure, LV end-diastolic pressure, dP/dt_max_ (maximum rate of increase of LV developed pressure), and dP/dt_min_ (maximum rate of decrease of LV developed pressure) between db/db and C57BL/6J mice at both 6 and 20 weeks of age (P > 0.05, n = 10 mice/group).

**Table 1:** LV hemodynamics of C57BL/6J and db/db mice.

	6 weeks old		6 weeks old	
	C57BL/6J	db/db	C57BL/6J	db/db
Heart rate, beats/min	427 ± 17	466 ± 18	456 ± 25	437 ± 20
LV end-systolic pressure, mmHg	94 ± 7	105 ± 7	101 ± 6	93 ± 6
LV end-diastolic pressure, mmHg	7 ± 1	6 ± 1	7 ± 1	9 ± 1
dP/dt_max_, mmHg/s	7344 ± 764	8605 ± 646	8513 ± 537	7600 ± 610
dP/dt_min_, mmHg/s	5926 ± 517	7032 ± 534	7257 ± 520	6010 ± 566

LV = left ventricular; s = second. There were no significant differences among groups in all parameters (n = 10 mice/group).

### Db/db mice at 20 weeks of age developed early DCM

The dimensions and function of the LV measured by echocardiography are shown in Table 2. LV anterior and posterior walls were increased in db/db mice at both 6 and 20 weeks of age compared with age-mated C57BL/6J mice (n = 12–13 mice/group, P < 0.05). There were no significant differences in LV internal diameters at both end diastole and end systole and fractional shortening between db/db mice and age-matched C57BL/6J mice (P > 0.05). Mitral E/A ratio was comparable between db/db and C57BL/6J mice at 6 weeks of age (P > 0.05, n = 12–13 mice/group). However, it was significantly lower in 20-week-old db/db mice than C57BL/6J controls (P < 0.05).

**Table 2:** Echocardiographic parameters of C57BL/6J and db/db mice.

	6 weeks old		20 weeks old	
	C57BL/6J	db/db	C57BL/6J	db/db
Anterior wall at end diastole, mm	0.80 ± 0.02	0.96 ± 0.03^*^	0.90 ± 0.04^*^	1.14 ± 0.03^*†#^
Anterior wall at end systole, mm	1.31 ± 0.04	1.54 ± 0.05^*^	1.26 ± 0.05^†^	1.63 ± 0.05^*#^
Posterior wall at end diastole, mm	0.80 ± 0.03	0.91 ± 0.04^*^	0.88 ± 0.03	1.16 ± 0.05^*†#^
Posterior wall at end systole, mm	1.18 ± 0.05	1.32 ± 0.04^*^	1.19 ± 0.05	1.46 ± 0.06^*#^
LV internal diameter at end diastole, cm	3.80 ± 0.08	3.71 ± 0.13	4.17 ± 0.09^*†^	3.78 ± 0.16
LV internal diameter at end systole, mm	2.25 ± 0.12	2.08 ± 0.14	2.88 ± 0.11^*†^	2.58 ± 0.11^†^
Fractional shortening, %	41 ± 3	45 ± 3	31 ± 1^*†^	31 ± 2^*†^
Peak E wave velocity, mm/s	682 ± 24	746 ± 29	573 ± 15^*†^	592 ± 28^*†^
Peak A wave velocity, mm/s	438 ± 22	499 ± 15^*^	351 ± 21^*†^	601 ± 38^*†#^
Peak E/A ratio	1.59 ± 0.09	1.50 ± 0.06	1.68 ± 0.08	1.00 ± 0.03^*†#^

LV = left ventricle; ms = millisecond. *P < 0.05 versus 6 week-old C57BL/6J mice; ^†^P < 0.05 versus 6 week-old db/db mice; and ^#^P < 0.05 versus 20 week-old C57BL/6J mice (n = 12–13 mice/group).

### Differently expressed LncRNAs in db/db mouse hearts with and without DCM

**Figure 2:**
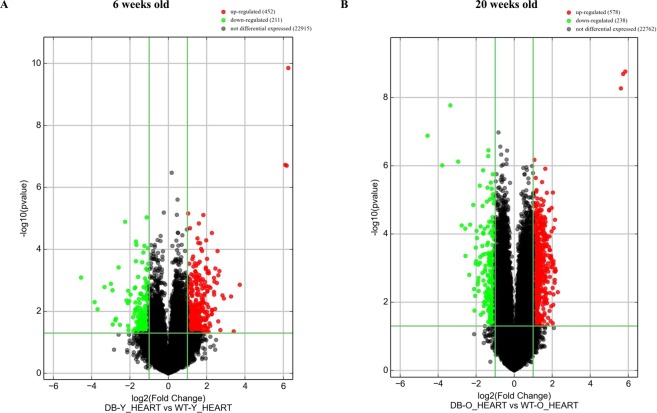
Volcano plots showing differently expressed lncRNAs in 6 and 20-week-old db/db mice, respectively compared with controls. The db/db mice developed early diabetic cardiomyopathy at 20 weeks old. The red and green points represented up- and down-regulated lncRNAs, respectively. The horizontal green line depicts *P ≤ 0.05, whereas the vertical green line shows a twofold change of up and down.

**Figure 3:**
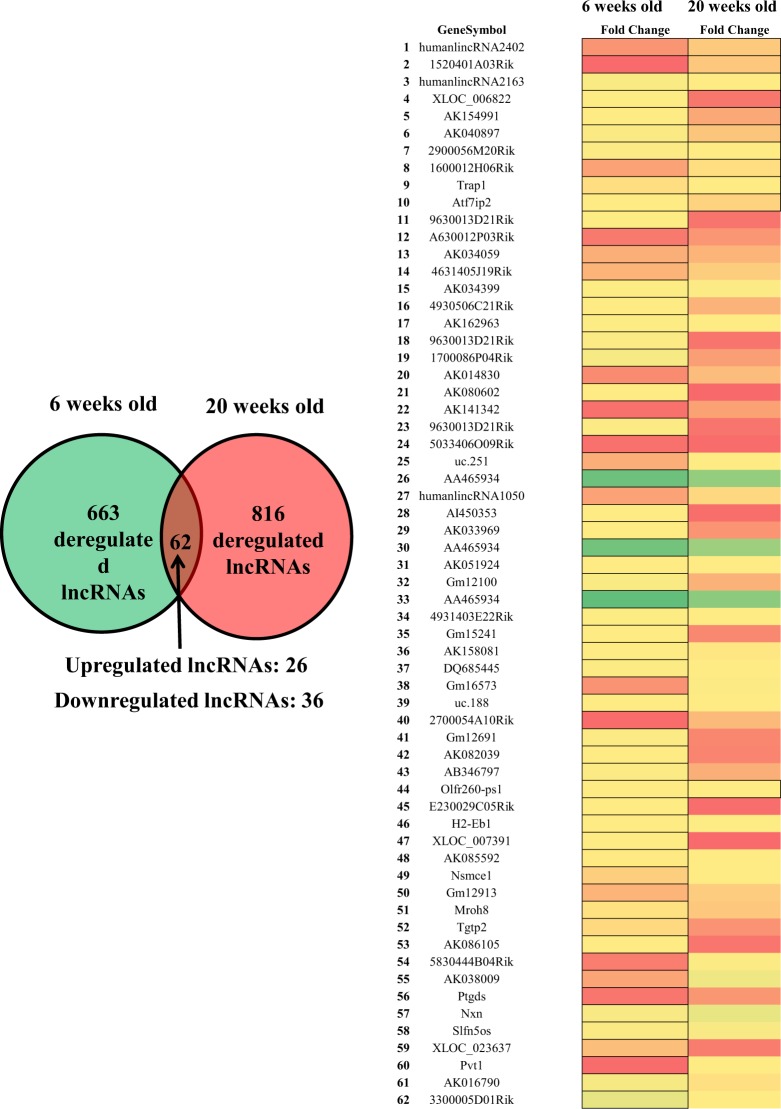
Overlapping deregulated lncRNAs in db/db mice between 6 and 20 weeks of age. Colors of red and green represented up- and down-regulated lncRNAs with changes larger than twofold, respectively.

**Figure 4:**
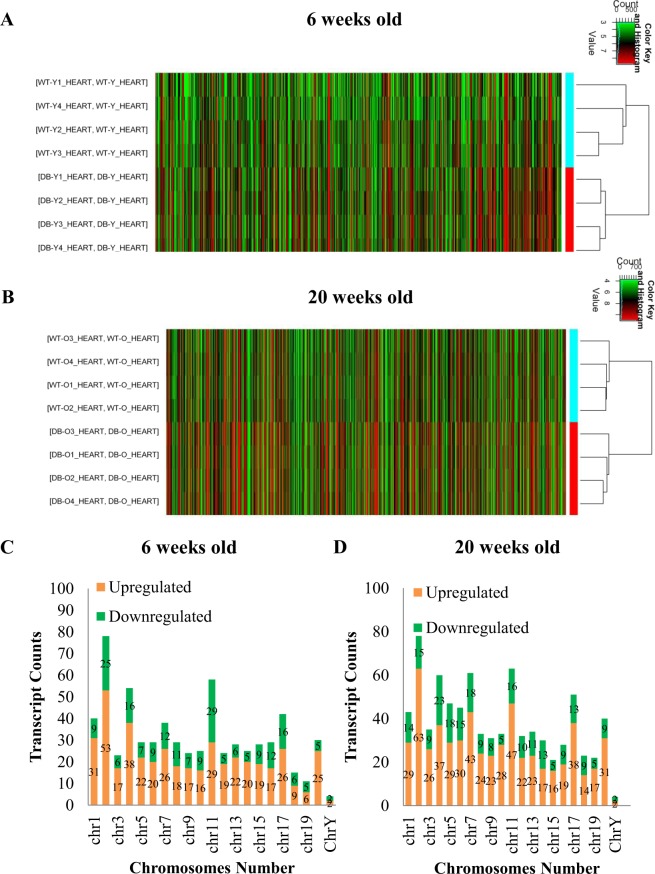
The profile of lncRNA expressions in db/db mouse hearts with and without early diabetic cardiomyopathy compared with controls. (**A**,**B**) hierarchical clustering analysis presenting differentially expressed lncRNAs between 6 and 20-week-old db/db and control mice, respectively. The db/db mice developed early diabetic cardiomyopathy at 20 weeks old. Colors of red and green represented up- and down-regulated lncRNAs with changes larger than twofold, respectively. (**C,D**) chromosomal distribution of deregulated lncRNAs in 6 and 20-week-old db/db mice, respectively. Colors of green and orange represented up- and down-regulated lncRNAs.

Figure 2 shows the profile of cardiac lncRNA expression in 6 and 20-week-old db/db mice compared with age-matched controls. Out of the total lncRNAs examined, 663 and 816 lncRNAs were deregulated in db/db mice at 6 and 20 weeks of age, respectively, compared with age-matched controls (Fig. 2A,B). Among them, 62 lncRNAs overlapped (Fig. 3). Compared with both 20-week-old C57BL/6J mouse hearts and 6-week-old db/db mouse hearts, 754 lncRNAs were deregulated in 20-week-old db/db mouse hearts. Hierarchical clustering analysis shows distinct lncRNA signature in young and older diabetic mice compared with controls (Fig. 4A,B).

The chromosomal distribution of deregulated lncRNAs was the same in young and older db/db mice (Fig. 4C,D). Chromosome 2 had the maximum number of deregulated lncRNAs in both young and older db/db mice. Compared with controls, 53 lncRNAs on chromosome 2 were up-regulated, constituting 11.7% of all deregulated lncRNAs (663), and other 25 lncRNAs were down-regulated in 6-week-old db/db mice. In 20-week-old db/db mice, 63 lncRNAs on chromosome 2 were up-regulated, constituting 9.58% of all deregulated lncRNAs (816), and 15 lncRNAs were down-regulated (Fig. 4C,D).

### Classification of differentially expressed lncRNAs in db/db mouse hearts

**Figure 5:**
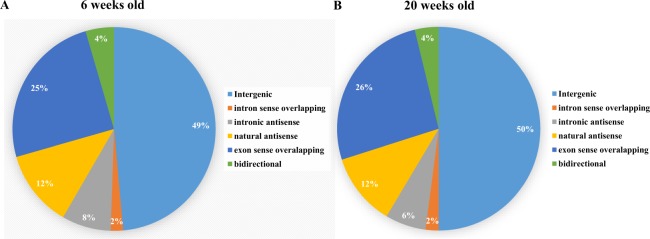
Classification of differently expressed lncRNAs in db/db mouse hearts with and without early diabetic cardiomyopathy. (**A**) percentage of lncRNA categories in 6-week-old db/db mouse hearts; (**B**) percentage of lncRNA categories in 20-week-old db/db mouse hearts.

Classification of differently expressed lncRNAs in db/db mouse hearts with and without DCM is shown in Fig. 5. Intergenic and sense lncRNAs accounted for approximately 50% and 30% of deregulated lncRNAs in both 6 and 20-week-old db/db mouse hearts, respectively. Antisense and bidirectional lncRNAs constituted about 15% and 5% of deregulated lncRNAs in both 6 and 20-week-old db/db mouse hearts, respectively.

### Differently expressed mRNAs in db/db mouse hearts with and without DCM

**Figure 6:**
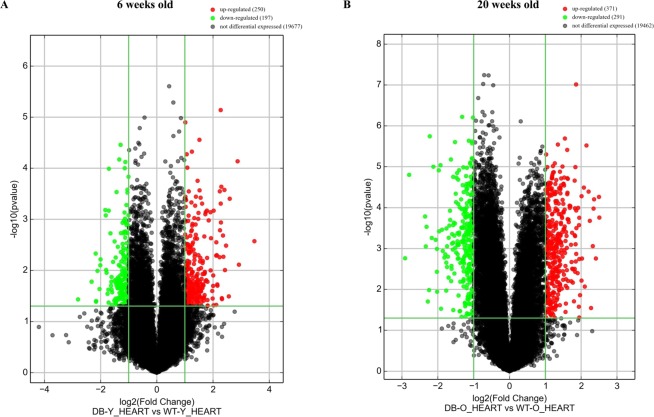
Volcano plots representing differently expressed mRNAs in 6 and 20-week-old db/db mice, respectively compared with age-matched controls. Db/db mice had early diabetic cardiomyopathy at 20 weeks old. The red and green points represented up- and down-regulated mRNAs, respectively. The horizontal green line depicts *P ≤ 0.05, whereas the vertical green line shows a twofold change of up and down.

**Figure 7:**
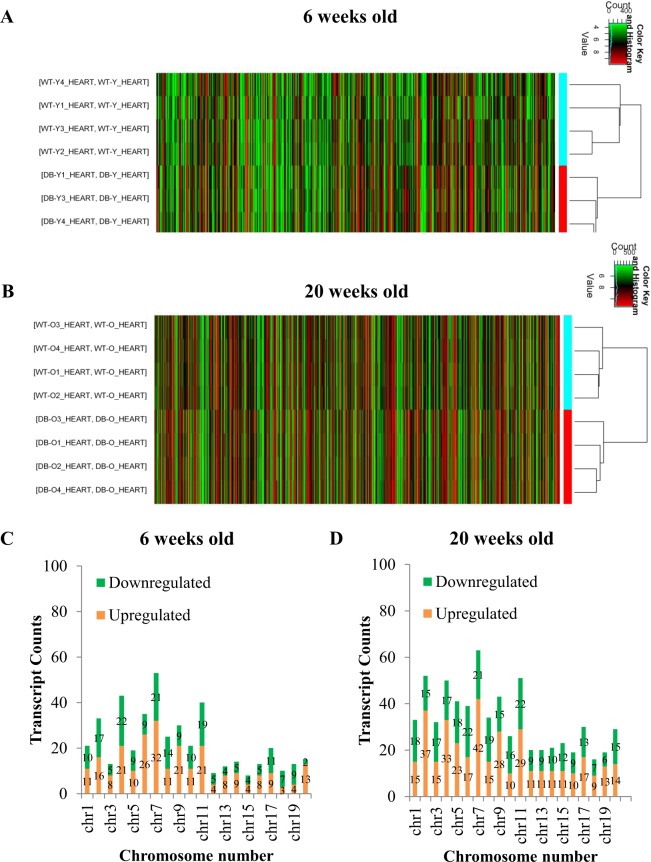
Profile of cardiac mRNA expression in db/db mouse hearts with and without early diabetic cardiomyopathy compared with controls. (**A**,**B**) hierarchical clustering analysis showing differently expressed mRNAs between 6 and 20-week-old db/db mice and controls, respectively. Colors of red and green represented up- and down-regulated lncRNAs with changes larger than twofold, respectively (**C**,**D**) chromosomal distribution of deregulated mRNAs in 6 and 20-week-old db/db mice, respectively. Colors of green and orange represented up- and down-regulated lncRNAs.

The profile of mRNA expression in db/db mouse hearts is shown in Fig. 6. A total of 24,881 mRNA transcripts were examined. Compared with controls, 250 and 371 mRNAs were up-regulated in 6 and 20-week-old db/db mice, respectively, and 197 and 291 mRNAs were down-regulated in 6 and 20-week-old db/db mice, respectively (Fig. 6A,B) (fold change ≥ 2.0 and P < 0.05). Hierarchical clustering analysis revealed different de-regulated mRNA signature in diabetic young and older mice compared with controls (Fig. 7A,B). Deregulated mRNAs distributed in 20 chromosomes in both 6 and 20 weeks-old db/db mice. Among 20 chromosomes, chromosome 7 had the maximum number of aberrantly expressed mRNAs (Fig. 7C,D). Compared with controls, 32 and 42 mRNAs in chromosome 7 were up-regulated in db/db mice at 6 and 20 weeks of age, respectively; whereas 21 mRNAs were down-regulated in db/db mice at both 6 and 20 weeks of age.

### Bioinformatics analysis of differentially expressed mRNAs in db/db mouse hearts with and without DCM

**Figure 8:**
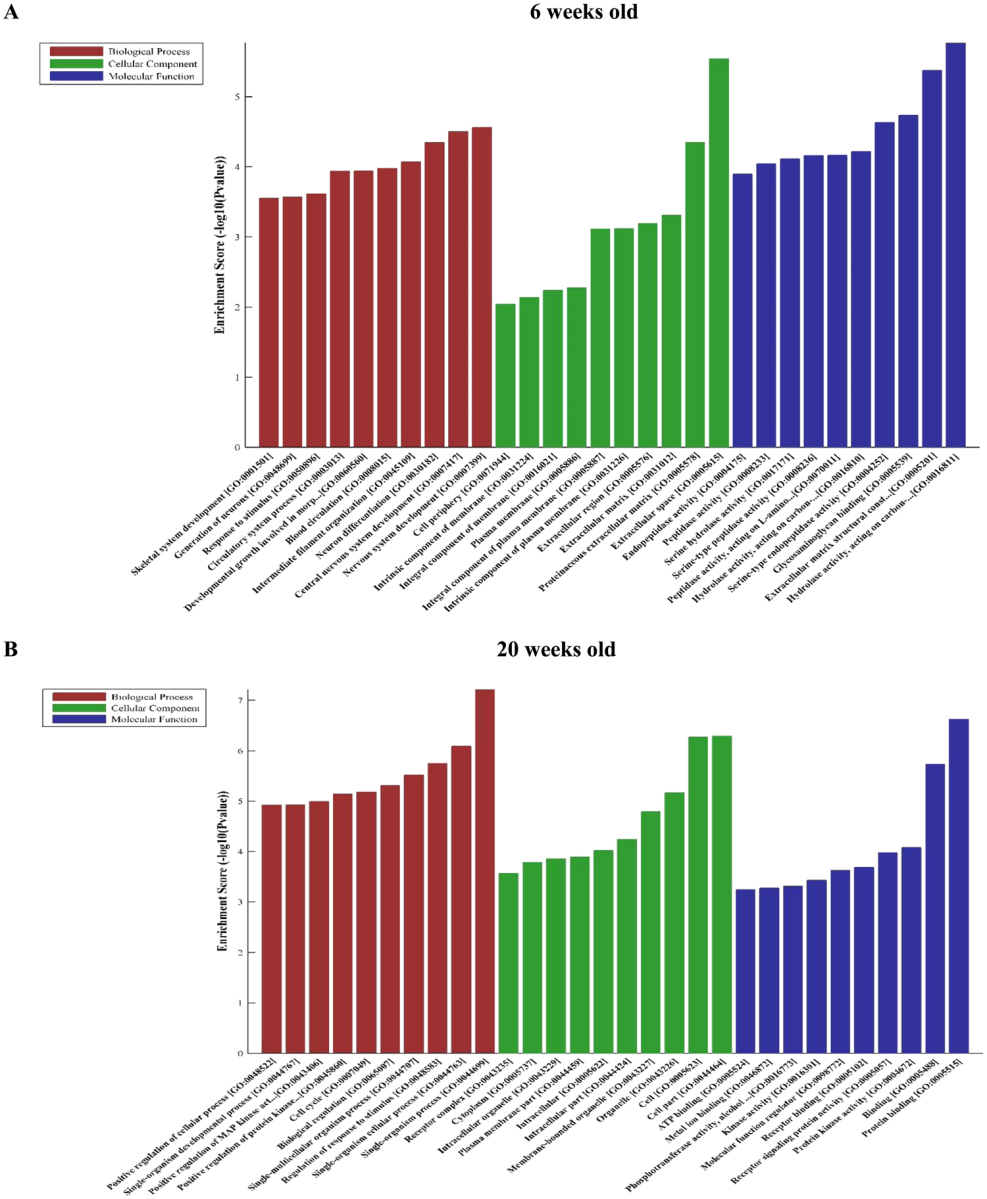
Significantly enriched gene ontology (GO) terms of top 30 up-regulated mRNAs in db/db mice at 6 and 20 weeks of age, respectively compared with controls.

**Figure 9:**
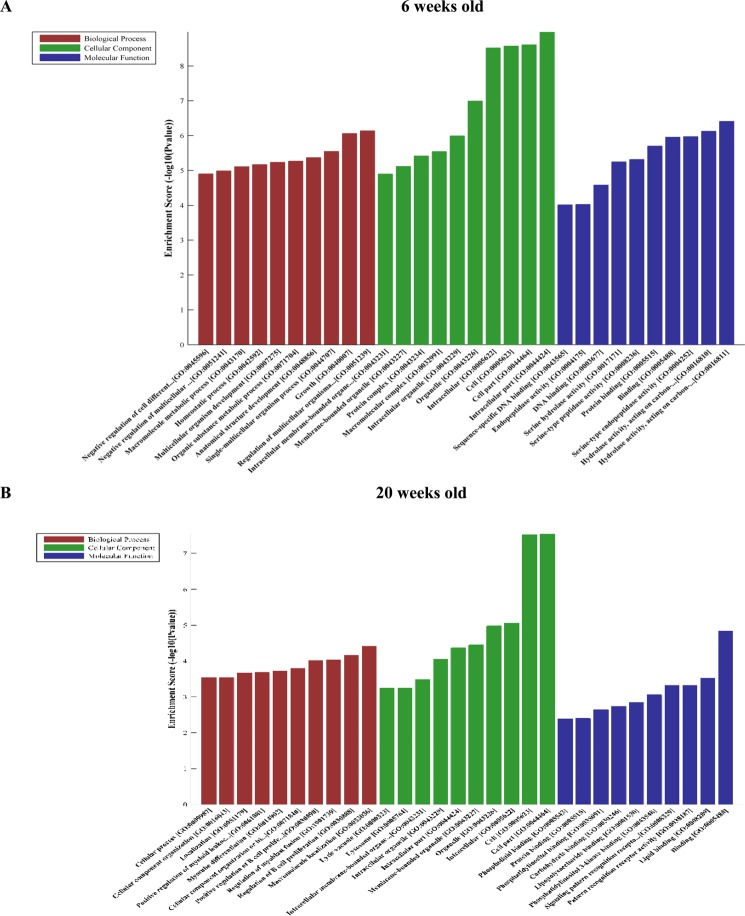
Significantly enriched GO terms of top 30 down-regulated mRNAs in db/db mice at 6 and 20 weeks of age, respectively compared with controls.

Figures 8 and 9 show GO analysis of 120 differentially expressed mRNAs in db/db mouse hearts with and without DCM. In 6-week-old db/db mouse hearts, top 30 up-regulated mRNAs were linked with 426 GO terms in the biological process network, 28 in cellular component networks, and 90 in molecular function network (Fig. 8A). In 20-week-old mouse hearts, top 30 up-regulated were associated with 529 GO terms in biological process network, 97 in cellular component network, and 112 in molecular function network (Fig. 8B). In 6-week-old db/db mouse hearts, the down-regulated mRNAs were associated with 926 GO terms in biological processes network, 67 in cellular component network, and 91 in molecular function network (Fig. 9A). In 20-week-old db/db hearts, the down-regulated mRNAs were linked 442 GO terms in biological processes network, 36 in cellular component network, and 96 in molecular function network (Fig. 9B).

**Figure 10:**
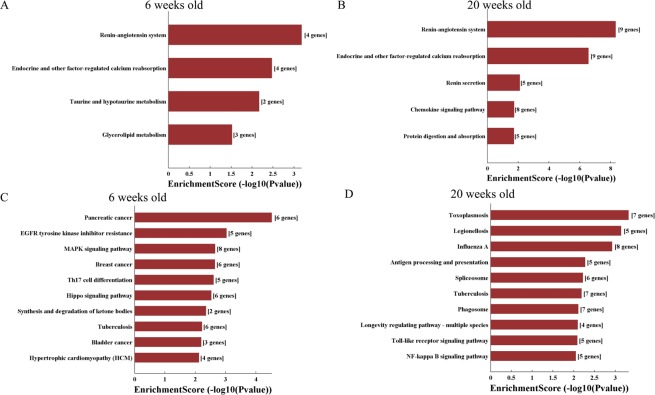
KEGG (Kyoto Encyclopedia of Genes and Genomes) pathway analysis of differentially expressed mRNAs in db/db mouse hearts with and without early diabetic cardiomyopathy. (**A**) the top 4 KEGG pathways of significantly up-regulated mRNAs between 6-week-old db/db and control mice; (**B**) the top 5 KEGG pathways of significantly up-regulated mRNAs between 20-week-old db/db and control mice; (**C**) the top 10 KEGG pathways of significantly down-regulated mRNAs between 6-week-old db/db and control mice; (**D**) the top 10 KEGG pathways of significantly down-regulated mRNAs between 20-week-old db/db and control mice. EGFR, epidermal growth factor receptors; MAPK: mitogen-activated protein kinase; NF-kB, nuclear factor kappa-light-chain-enhancer of activated B cells.

Figure 10 shows the KEGG pathway analysis of deregulated mRNAs in db/db mouse hearts. In 6-week-old db/db diabetic hearts, up-regulated mRNAs were significantly enriched for renin-angiotensinogen system, endocrine and other factor-regulated Ca^2+^ reabsorption, and taurine and hypotaurine metabolism (Fig. 10A). In 20-week-old db/db diabetic hearts, up-regulated mRNAs were enriched for renin-angiotensinogen system, endocrine and other factor-regulated Ca^2+^ reabsorption, and chemokine signaling pathway (Fig. 10B). In 6-week-old db/db diabetic hearts, down-regulated mRNAs were significantly enriched for mitogen-activated protein kinase signaling pathway, pancreatic cancer, and breast cancer (Fig. 10C). In 20-week-old db/db diabetic hearts, down-regulated mRNAs were enriched for influenza A, toxoplasmosis, and tuberculosis (Fig. 10D).

### LncRNA-mRNA co-expression networks

**Figure 11:**
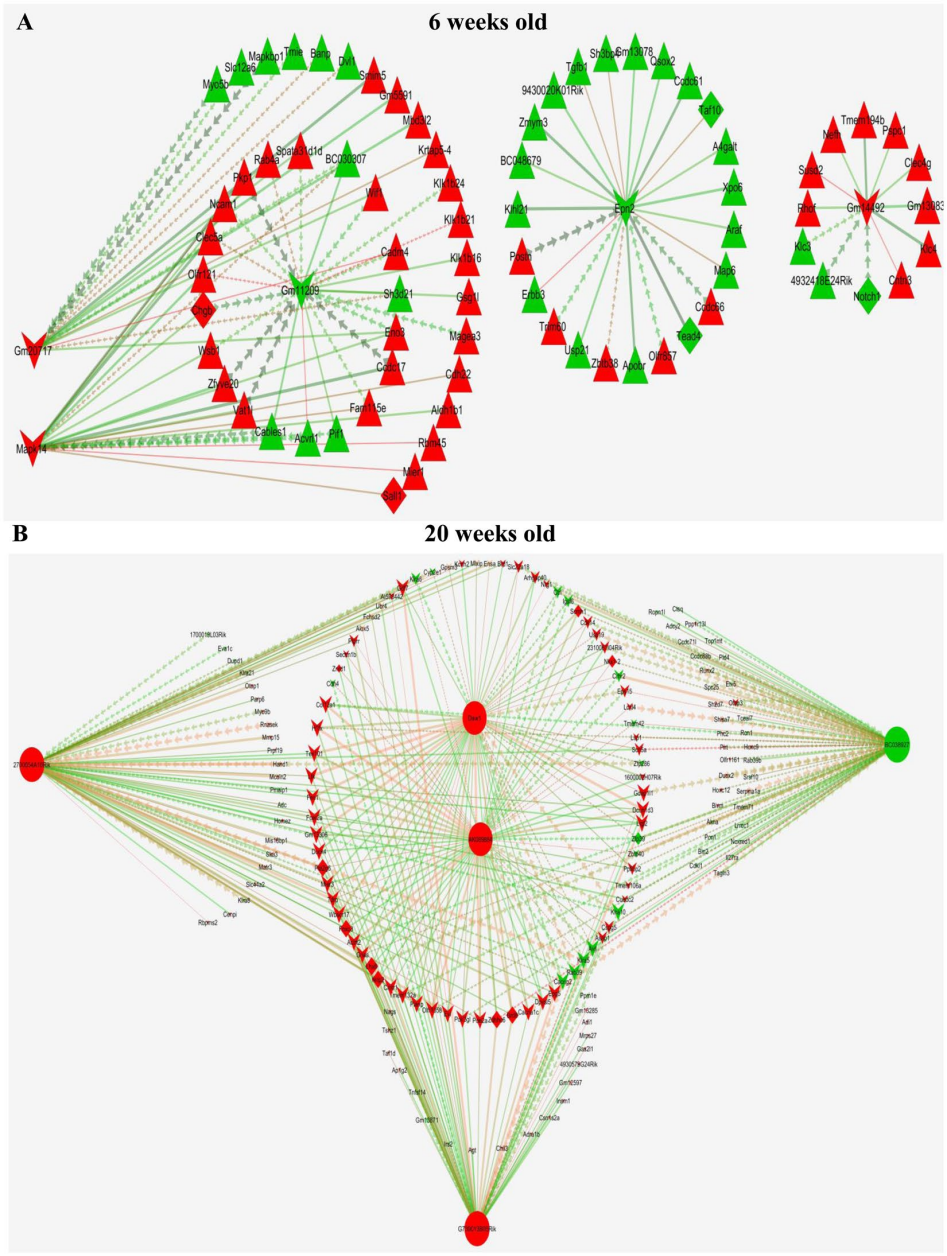
Correlation between significantly differentially expressed lncRNAs and mRNA transcripts. (**A**) the network of lncRNA-mRNA co-expression in 6-week-old db/db mice; (**B**) the network of lncRNA-mRNA co-expression in 20-week-old db/db mice. The network represents the co-expression correlations between the significantly differentially expressed lncRNAs and mRNA transcripts. Five lncRNAs having maximum connections with mRNAs were taken to construct the co-expression network (Pearson correlation >0.995 or <−0.995 and P < 0.05). Circles, squares, and V shapes indicate lncRNA transcripts, transcription factors, and mRNA transcripts, respectively. Solid arrows and dashed lines indicate positive and negative correlation, respectively, whereas red and green colors represent up- and down-regulated transcripts. The width of the line is based on Person’s value (stronger correlation corresponds to more width) and color of the line depicts the significance. Nodes indicated lncRNAs or mRNAs.

**Figure 12:**
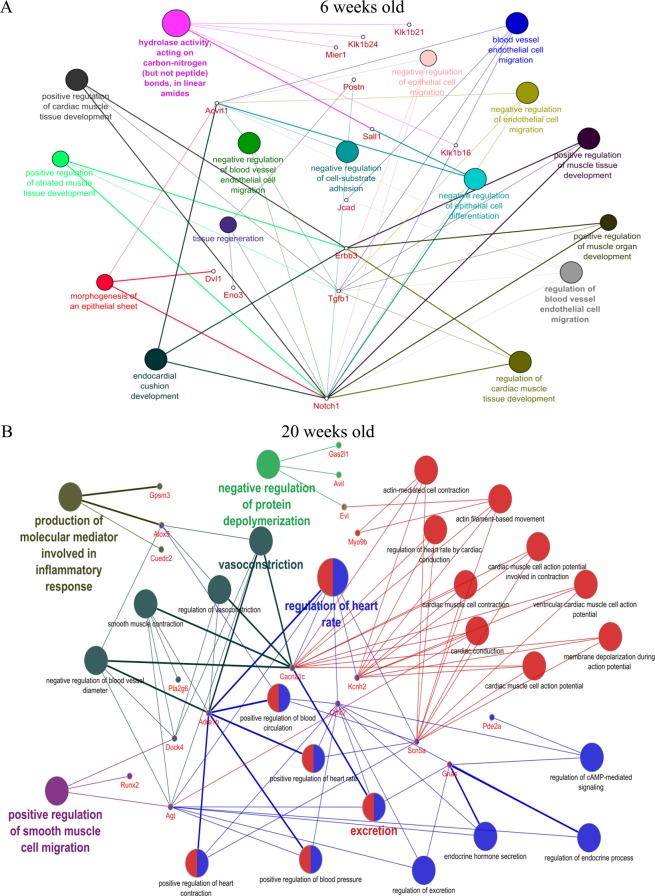
Gene ontology (GO) analysis of the 5 deregulated lncRNAs that had maximum connections with mRNAs. (**A**) The network represents the GO pathway terms specific for mRNA genes having co-expression relationship with the significantly differentially expressed top 5 lncRNAs having maximum connections in the Co-expression network (Pearson correlation >0.995 or <−0.995 and P < 0.05) at 6 weeks of age; (**B**) the network represents the GO pathway terms specific for mRNA genes having co-expression relationship with the significantly differentially expressed top 5 lncRNAs having maximum connections in the Co-expression network (Pearson correlation >0.995 or <−0.995 and P < 0.05) at 20 weeks of age. Functionally grouped networks with GO terms as node are grouped on the basis of kappa score level (>0.03), the most significant groups are shown. Each node represents a GO biological process, and the colors represent the GO group. The node size represents the term enrichment significance. Different GO clusters are shown in different color. The edges reflect the relationships between the terms based on the similarity of their associated genes.

Significantly co-expressed lncRNAs-mRNAs (Pearson correlation > 0.995 or < −0.995 and P < 0.05) were identified and assembled into co-expression networks. There were 10,100 connections between 654 lncRNAs and 439 mRNAs in db/db mouse hearts at 6 weeks of age and 21,463 connections between 1601 lncRNAs and 1277 mRNAs in db/db mouse hearts with DCM. Figure 11 shows the co-expression network of 5 lncRNAs having the maximum number of connections with mRNAs. In 6-week-old db/db mouse hearts, top 5 lncRNAs (Gm20717, Gm14492, Gm11209, Epn2, Mapk14) were connected with 76 mRNAs (Fig. 11A). These 5 lncRNAs were correlated with the development of myocardial tissues (GO: 0055024), positive regulation of myocardial development (GO: 1901863), positive regulation of striated muscle tissue development (GO: 0045844), positive regulation of muscle organ development (GO: 0048636), tissue regeneration (GO: 0042246), and hydrolase activity (GO: 0016811) (Pearson correlation > 0.995 or < −0.995 and P < 0.005) (Fig. 12A). In 20-week-old db/db mouse hearts, top 5 network nodes (BC038927, G730013B05Rik, 2700054A10Rik, AK089884, Daw1) were linked with 126 mRNAs (Fig. 11B). These lncRNAs were correlated with action potential (GO: 0086001), membrane depolarization (GO: 0070252), conduction (GO: 0061337), contraction (GO: 0086003), and actin filament based movement (GO: 0030048) of cardiac muscles (Pearson correlation > 0.995 or < −0.995 and P < 0.005) (Fig. 12B).

### Validation of lncRNAs and mRNAs using quantitative reverse transcriptional-polymerase chain reaction

To validate the RNA-sequence data, we used quantitative reverse transcriptional-polymerase chain reaction (qRT-PCR) to analyze 12 lncRNAs and 12 mRNAs in 6 and 20-week-old mouse hearts. qRT-PCR analysis revealed that the expression levels of the lncRNAs, AA465934, Ccdc92, AK030243, AA465934, Sart3, and Meg3, were up-regulated in db/db mouse hearts, while those of XLOC_010800, 3300005D01Rik, Gm12913, AK143388, Nxn, and AK038009 were down-regulated in db/db mouse hearts. Similarly, the expression levels of the mRNAs, Klk1b16, AI593442, Fbxw19, AI593442, Klk1b16, and Cmtr1 were up-regulated in db/db mouse hearts, whereas those of Nkd1, Ttc39a, Cd74, Gdf15, 5031410I06Rik, and Gm10220 were down-regulated in db/db mouse hearts. The qRT-PCR results of both lncRNAs and mRNAs were mostly consistent with the microarray data.

## Discussion

The results of the present study demonstrate that db/db mice have diabetes, obesity, and LV hypertrophy at 6 weeks of age and develop early DCM at 20 weeks of age. The profiling of both lncRNA and mRNA expression is different between db/db mouse hearts without DCM and with early DCM. We identified 754 lncRNAs that were aberrantly expressed in db/db mouse hearts with early DCM. Among them, the lncRNAs, BC038927, G730013B05Rik, 2700054A10Rik, AK089884, and Daw1, have maximum connections with differentially expressed mRNAs. Bioinformatics analysis reveals that these 5 lncRNAs are closely associated with membrane depolarization, action potential conduction, contraction, and actin filament-based movement of cardiac cells. These results suggest that BC038927, G730013B05Rik, 2700054A10Rik, AK089884, and Daw1, may be the core lncRNA with high significance.

The db/db mouse is the most widely used rodent models in obesity-induced T2DM research^18,19^. Db/db mice suffer from a deficiency or mutation of leptin receptors on chromosome 4^20^. Leptin is an adipose tissue hormone and functions to regulate food intake, energy homeostasis, thermogenesis, reproduction, hematopoiesis, skeletal growth, and neuroprotection by activating the cognate leptin receptors^21^. In the db/db mouse, leptin fails to exert its actions due to the deficiency or mutation of leptin receptors, displaying hyperglycemia, obesity, hyperinsulinemia, and dyslipidemia^17^. This was confirmed by our present study showing that the db/db mice at both 6 and 20 weeks of age had increased body weight, blood glucose, and plasma insulin levels.

In humans and monkeys, DCM is characteristic of cardiac (both diastolic and later systolic) dysfunction that occurs independently of a recognized cause such as coronary artery disease or hypertension^8,22,23^. In the present study, db/db mice at 6 weeks of age had normal cardiac function, despite hyperglycemia, obesity, and cardiac hypertrophy. These results are consistent with our previous report in db/db mice with the genetic background of C57BLKS/J (C57BL/KsJ-lepr^*db*^/lepr^*db*^)^18^. Intriguingly, the db/db mice at 20 weeks of age had normal systolic function (fractional shortening) but decreased diastolic function (mitral E/A ratio). This diastolic dysfunction was independent of LV pressure. Moreover, the electrocardiogram examination of 20-week-old db/db mice revealed that there was no myocardial ischemia (data not shown). Thus, depressed diastolic function in 20-week-old db/db mice can be attributed to early DCM.

A growing number of studies indicate that lncRNAs play crucial roles in various cardiovascular diseases, including cardiac hypertrophy, heart failure, and diabetic vascular complications^24–28^. Recent studies reported that a few lncRNAs, including H19, metastasis-associated lung adenocarcinoma transcript 1 (MALAT1), and myocardial infarction-associated transcript (MIAT), were involved in the pathogenesis of DCM in type 1 diabetic animals^16,29,30^. We identified 754 lncRNAs that were differentially expressed in diabetic db/db mouse hearts with early DCM. It is likely that multiple deregulated lncRNAs contribute to the pathogenesis of early DCM in the T2DM db/db mice.

In the present study, 754 deregulated lncRNAs in db/db mouse hearts with early DCM were distributed on 21 chromosomes. However, these lncRNAs were not equally distributed across all chromosomes. Compared with other chromosomes, chromosome 2 had the maximum number of deregulated lncRNAs in db/db mouse hearts with and without DCM. Diabetic cardiovascular disease is increasingly identified to associate with genetic susceptibility^31–34^. Therefore, chromosome 2 may be more likely to carry lncRNAs susceptible to the pathology of DCM.

LncRNAs regulate epigenetically the expression of broad ranges of genes in cardiomyocytes^13,35^. To gain a better insight into the biological function of deregulated lncRNAs and conduct the analysis of transcribed sequences, it is advantageous to arrange them into several clusters^4,36^. Based on the relationship between lncRNAs and their affiliated protein-coding genes, lncRNAs detected by Arraystar Array are characterized as antisense, bidirectional, intronic, sense overlapping and intergenic lncRNAs^36^. The majority of differentially expressed lncRNAs in our study were intergenic (50%) and antisense (30%), which contributes to more than three-quarters of the deregulated lncRNAs. LncRNAs are transcribed from regions of at least 5 kb, from protein-coding genes. They can modulate the expression of target genes, and the target genes can be scattered across the genome. Antisense lncRNAs are capable of altering the expression of protein-coding genes and affect their protein-coding counterparts through various mechanisms^37^. The many abnormally expressed intergenic lncRNAs and antisense lncRNAs in DCM indicate that lncRNAs may regulate protein-coding genes throughout the development of DCM.

Our microarray data showed that a large number of mRNAs were aberrantly regulated in db/db mouse hearts with and without DCM. Altogether, 447 and 662 mRNAs were found to differentially express in db/db hearts without DCM and db/db hearts with DCM, respectively. Overall, more mRNAs were up-regulated than down-regulated. Deregulated mRNAs were distributed on 21 chromosomes, among which chromosome 7 had the maximum number of aberrantly expressed mRNAs.

LncRNAs are increasingly identified to play roles in a broad variety of principal biologic activities^35,38^. The function of lncRNAs proximately associates with their correlated protein-coding genes, thus studying the genomic background of lncRNAs can help predict their essential biological function^39^. Based on the GO and KEGG database analyses of lncRNAs, protein-coding genes, and mRNAs, up-regulated genes were significantly enriched for renin-angiotensinogen system, endocrine and other factor-regulated Ca^2+^ reabsorption, and taurine and hypotaurine metabolism in db/db diabetic hearts without DCM and for renin-angiotensinogen system, endocrine and other factor-regulated Ca^2+^ reabsorption, and chemokine signaling pathway in db/db diabetic hearts with DCM. Whereas down-regulated mRNAs were significantly enriched for mitogen-activated pancreatic cancer, protein kinase signaling pathway, and breast cancer in db/db diabetic hearts without DCM and for influenza A, toxoplasmosis, and tuberculosis in db/db diabetic hearts with DCM. Previous studies have shown that cancers, viral infections, and tuberculosis are higher in diabetic patients^40–43^. Thus, these down-regulated mRNAs in diabetes may be involved in increased cancers and infection.

The integrated analysis of the lncRNA-mRNA co-expression networks revealed that 5 deregulated lncRNAs having maximum connections with differentially expressed genes were BC038927, G730013B05Rik, 2700054A10Rik, AK089884, and Daw1. Functional analysis identified these 5 lncRNAs were correlated with membrane depolarization of cardiac muscles like actin mediated cell contraction (GO: 0070252), cardiac muscle cell contraction (GO: 0086003), cardiac conduction (GO: 0061337), cardiac muscle cell action potential (GO: 0086001), and actin filament based movement (GO: 0030048). To our knowledge, there are no previous studies reporting the function and role of these lncRNAs in DCM. However, our results suggest that these 5 lncRNAs may be core lncRNA with high significance for early DCM.

In summary, the results of our investigation indicate that db/db mice at 20 weeks of age develop early DCM. Among aberrant expression of the 754 lncRNAs that are associated with the pathogenesis of early DCM, BC038927, G730013B05Rik, 2700054A10Rik, AK089884, and Daw1, have maximum associations with mRNAs. Given that these 5 lncRNAs are associated with membrane depolarization, action potential conduction, contraction, and actin filament-based movement of cardiac cells, they may serve as important therapeutic targets for DCM.

## Materials and Methods

### Animals

Obese male db/db and C57BL/6J control mice were purchased from The Jackson Laboratory (Bar Harbor, ME, USA). The animals were kept on a 12-h light-dark cycle in a temperature-controlled room. The animal care and all experimental procedures were performed in accordance with the NIH *Guide for the Care and Use of Laboratory Animals* (Institute for Laboratory Animal Research, National Academy of Sciences, 8th edition, 2011), and experimental protocols were approved by the Institutional Animal Care and Use Committee (IACUC) at the Medical College of Wisconsin (Milwaukee, WI, USA).

Our pilot experiments showed the db/db mouse had the normal systolic function of the left ventricle but started to have impaired diastolic function at 20 weeks of age. Based on the preliminary studies, the db/db mice were divided into 2 experimental groups: diabetic mice without DCM (db/db mice at 6 weeks of age) and diabetic mice with early DCM (db/db mice at 20 weeks of age). Age- and gender-matched C57BL/6J mice were used as controls.

### Measurements of blood glucose and insulin

C57BL/6J and db/db mice at either 6 or 20 weeks of age were fasted for 6 h (12–13 mice/group). Under the anesthesia of 80 mg/kg pentobarbital, a thoracotomy was performed, and the left ventricle was punctured with a 27 gauge needle, as described previously^44^. Blood glucose was measured with a blood gas analyzer (ABL-725 Radiometer, Radiometer America Inc., Westlake, OH, USA)^45^. In ice‐chilled heparinized tubes, plasma was immediately separated and stored frozen at −80 °C. Plasma insulin levels were measured by radioimmunoassay in 20 μl aliquots of plasma using a commercial kit (Linco Research Inc., St Charles, MI, USA)^46^.

### LV hemodynamic measurement

C57BL/6J and db/db mice were anesthetized by the inhalation of 2.0% isoflurane and oxygen (n = 10 mice/group). The mice were ventilated with room air supplemented with 100% oxygen at approximately 102 breaths/min, as described^44,47^. The right carotid artery was cannulated with a Millar Mikro-Tip Pressure Transducer Catheter (1.4-Fr, model SPR 671, Millar Instruments, Inc.; Houston, TX, USA), and the catheter was placed in the middle of the LV chamber to measure left ventricular systolic and diastolic pressure, as described^48^. Before every measurement of left ventricular pressure (LVP), the catheter was calibrated electronically *in vitro* by submerging its tip in the saline solution of 37 °C and applying an external pressure of 100 mmHg. The catheter was connected to an ADInstrument pressure transducer (MLT0380/D, ADInstruments, Colorado Springs, CO, USA) and a Powerlab data acquisition system (ADInstruments). After a 30 min of stabilization, blood pressure was continuously recorded for 20 min^48^. The LVP signal was monitored, and dP/dt_max_ and dP/dt_min_ were determined. Body temperature was maintained between 36.8 °C and 37.3 °C throughout the experiment by using a heating pad (Model TC-1000, CWE Inc.; Ardmore, PA, USA).

### Transthoracic echocardiography

C57BL/6J mice and db/db mice were sedated by the inhalation of 1.50% isoflurane and oxygen (n = 12–13 mice/group). Non-invasive transthoracic echocardiography was performed with a VisualSonics Vevo 770 High-resolution Imaging System (Toronto, Canada) equipped with a 30 MHz transducer (Scanhead RMV 707), as described^18,49^. LV dimensions and fractional shortening were measured by two-dimension guided M-mode method. Pulsed Doppler waveforms recorded in the apical-4-chamber view were used for the measurements of the peak velocities of mitral E (early mitral inflow) and A (late mitral inflow) waves.

### RNA extraction

After echocardiographic examination was completed, the C57BL/6J and db/db mice were euthanized to harvest the LV (n = 12–13 mice/group). Myocardial tissues were immersed immediately into liquid nitrogen, and total RNAs were isolated using Triazol Reagent (Invitrogen, Carlsbad, CA, USA), as described^50,51^. The extracts were further quantified using Nano Drop ND-1000. Presence of DNA contaminants was assessed by agarose gel electrophoresis on a denaturing gel, whereas RNA integrity was determined using Bioanalyzer 2100 (Agilent Technologies, Santa Clara, CA, USA). Array hybridization was performed, according to the Agilent One-Color Microarray-Based Gene Expression Analysis protocol (Agilent Technologies) with minor modifications. Briefly, mRNA was purified from total RNAs after removal of rRNA with mRNA-ONLY™ Eukaryotic mRNA Isolation Kit (Epicentre Biotechnologies, Madison, WI, USA). Each sample was amplified and transcribed into fluorescent cRNA along the entire length of the transcripts without 3ʹ bias utilizing a mixture of oligo(dT) and random priming method with Arraystar Flash RNA Labeling Kit (Arraystar Inc., Rockville, MD, USA). The labeled cRNA was purified by RNeasy Mini Kit (Qiagen, Valencia, CA, USA). The concentrations and specific activity of the labeled cRNA (pmol Cy3/μg cRNA) were measured by Nano Drop ND-1000. One μg of each labeled cRNA was fragmented by adding 5 μl 10× blocking agent and 1 μl of 25× Fragmentation Buffer, then heated the mixture at 60 °C for 30 min, finally 25 μl 2 × GE Hybridization buffer was added to dilute the labeled cRNA. Fifty μl of hybridization solution was dispensed into the gasket slide and assembled to the lncRNA expression microarray slide. The slides were incubated for 17 h at 65 °C in an Agilent hybridization oven. The hybridized arrays were washed, fixed, and scanned by the Agilent DNA Microarray Scanner (Agilent Technologies).

### Microarray and bioinformatics analysis

The profiling of lncRNA and mRNA expression was performed using Arraystar Mouse LncRNA Microarray V3.0 (Arraystar Inc.), which detected 35,923 lncRNAs and 24,881 mRNAs^52^. Agilent Feature Extraction software version 11.0.1.1 (Agilent Technologies) was used to analyze acquired array images. Quantile normalization and subsequent data processing were performed using the GeneSpring GX v12.1 software package (Agilent Technologies). After quantile normalization of the raw data, lncRNAs and mRNAs that at least 4 out of 16 samples had flags in Present or Marginal (“All Targets Value”) were chosen for further data analysis. Statistically significant changes between the two groups were considered when the fold changes for differentially expressed lncRNAs and mRNAs were larger than 2.0, and the P value for *t*-test was less than 0.05. Differentially expressed lncRNAs and mRNAs with statistical significance were identified through Volcano Plot filtering between two groups. Hierarchical clustering was performed using the R software (version 2.15). GO (www. geneontology.org) and KEGG enrichment analyses were made to annotate the potential functions of differentially expressed lncRNAs. To identify the potential transcriptional regulators, the analysis of upstream regulators was conducted using the Ingenuity Pathway Analysis software (Ingenuity Systems Inc., Redwood City, CA, USA). Only transcriptional regulators that appeared up- or down-regulated in the microarray study were considered for the analysis.

### LncRNA-mRNA correlation analysis

To reveal potential association of the differentially expressed lncRNAs with mRNAs in DCM, we built the lncRNA-mRNA co-expression networks using Cytoscape software (v3.4.0), according to the normalized signal intensity of individual genes^53^. Briefly, microarray data were pre-processed by using the average expression value of all transcripts expressed from the same gene (both mRNA and lncRNA). The data were then screened for differentially expressed lncRNAs and mRNAs whose expression levels positively or negatively correlated. For each pair of lncRNA-mRNA, *Pearson* correlation test was conducted to detect significant correlation. Only strongly correlated (r^2^ ≥ −0.9, P* < *−0.01) pairs were used to construct the networks and generate visual representations. In these representations, each gene corresponded to a node, and the color of nodes represented the up-regulated or down-regulated expression of the specific gene in the microarray data.

### qRT-PCR reaction analysis of lncRNAs and mRNAs

To confirm the reliability of the lncRNA and mRNA microarray data, we selected 12 lncRNAs and 12 mRNAs that exhibited significant changes (FCs > 6.0) for validation using qRT-PCR^45,51^. Three up-regulated lncRNAs were AA465934, Ccdc92, and AK030243 in 6-week-old db/db mouse hearts and AA465934, Sart3, and Meg3 in 20-week-old db/db mouse hearts, and three down-regulated lncRNAs were lncRNAs XLOC_010800, 3300005D01Rik, and Gm12913 in 6-week-old db/db mouse hearts and AK143388, Nxn and AK038009 in 20-week-old db/db mouse hearts. Three up-regulated mRNAs were Klk1b16, AI593442, Fbxw19 in 6-week-old db/db mouse hearts and AI593442, Klk1b16, Cmtr1 in 20-week-old db/db mouse hearts. Three down-regulated mRNAs were Nkd1, Ttc39a, and Cd74 in 6-week-old db/db mouse hearts and Gdf15, 5031410I06Rik, and Gm10220 in 20-week-old db/db mouse hearts.

Total RNA from the LV was extracted using Triazol Reagent, as described above. Chloroform was added, and samples were centrifuged to facilitate phase separation. The aqueous phase was extracted and combined with ethanol in miRNeasy Mini spin columns (Qiagen). Total RNA was eluted in RNase-free water, and the concentration of extracted total RNA was quantified by the Epoch spectrophotometer (Biotek, Winooski, VT). Samples were considered pure if the A260/280 ratio was between 1.9 and 2.0. One µg of total RNA from each sample was used to generate cDNA using miScript Reverse transcriptase mix, nucleic mix, and HiFlex Buffer (Qiagen). To analyze the lncRNA expression, a master mix (25 μl/well) containing the template cDNA (4.5 ng/well), RNase-free water, and miScript SYBR Green (Qiagen), and the primers (lncRNAs or the housekeeping gene, Rnu-6) was prepared according to the manufacturer’s directions. Quantitative reverse transcriptional-polymerase chain reaction (qRT-PCR) was conducted using the BioRad iCycler Real-Time PCR Detection System^50^. qRT-PCR for each sample was run in triplicate. Expression of lncRMAs was normalized by expression of Rnu-6. The relative gene expressions were calculated in accordance with the ΔΔCt method.

### Statistical analysis

Kruskal-Wallis test followed by Dunn’s test was used to compare body weight, blood glucose, and insulin levels. Repeated-measures analysis of variance followed by Bonferroni multiple comparison test was used to evaluate differences in LV hemodynamic data and echocardiographic data. Non-parametric Mann Whitney test was used to compare the gene expression between two groups, whereas Benjamini-Hochberg FDR (cut off 0.05) was applied for multiple-testing correction. All statistical analyses were performed using GraphPad Prism 8 (GraphPad Software, Inc., La Jolla, CA, USA). A value of P less than 0.05 (two tailed) was considered statistically significant.
